# Epistasis of HTR1A and BDNF risk genes alters cortical 5-HT1A receptor binding: PET results link genotype to molecular phenotype in depression

**DOI:** 10.1038/s41398-018-0308-2

**Published:** 2019-01-16

**Authors:** Alexander Kautzky, Gregory M. James, Cecile Philippe, Pia Baldinger-Melich, Christoph Kraus, Georg S. Kranz, Thomas Vanicek, Gregor Gryglewski, Annette M. Hartmann, Andreas Hahn, Wolfgang Wadsak, Markus Mitterhauser, Dan Rujescu, Siegfried Kasper, Rupert Lanzenberger

**Affiliations:** 1 Department of Psychiatry and Psychotherapy, Medical University of Vienna, Wien, Austria; 2 Division of Nuclear Medicine, Department of Biomedical Imaging and Image-guided Therapy, Medical University of Vienna, Wien, Austria; 3 University Clinic for Psychiatry, Psychotherapy and Psychosomatic, Martin-Luther-University Halle-Wittenberg, Halle, Germany; 4 Center for Biomarker Research in Medicine (CBmed), Graz, Austria; 5 Ludwig Boltzmann Institute Applied Diagnostics, Vienna, Austria

**Keywords:** Depression, Molecular neuroscience, Genomics

## Abstract

Alterations of the 5-HT_1A_ receptor and BDNF have consistently been associated with affective disorders. Two functional single nucleotide polymorphisms (SNPs), rs6295 of the serotonin 1A receptor gene (*HTR1A*) and rs6265 of brain-derived neurotrophic factor gene (*BDNF*), may impact transcriptional regulation and expression of the 5-HT_1A_ receptor. Here we investigated interaction effects of rs6295 and rs6265 on 5-HT_1A_ receptor binding. Forty-six healthy subjects were scanned with PET using the radioligand [*carbonyl-*^11^C]WAY-100635. Genotyping was performed for rs6265 and rs6295. Subjects showing a genotype with at least three risk alleles (G of rs6295 or A of rs6265) were compared to control genotypes. Cortical surface binding potential (BP_ND_) was computed for 32 cortical regions of interest (ROI). Mixed model was applied to study main and interaction effects of ROI and genotype. ANOVA was used for post hoc analyses. Individuals with the risk genotypes exhibited an increase in 5-HT_1A_ receptor binding by an average of 17% (mean BP_ND_ 3.56 ± 0.74 vs. 2.96 ± 0.88). Mixed model produced an interaction effect of ROI and genotype on BP_ND_ and differences could be demonstrated in 10 ROI post hoc. The combination of disadvantageous allelic expression of rs6295 and rs6265 may result in a 5-HT_1A_ receptor profile comparable to affective disorders as increased 5-HT_1A_ receptor binding is a well published phenotype of depression. Thus, epistasis between *BDNF* and *HTR1A* may contribute to the multifactorial risk for affective disorders and our results strongly advocate further research on this genetic signature in affective disorders.

## Introduction

The monoamine neurotransmitter serotonin has an essential role in behavior and cognition^1^. Especially for affective disorders serotonin is regarded as the decisive neurotransmitter, implicated in the etiology and course of the most common neuropsychiatric diseases major depressive disorder (MDD) and anxiety disorders^2,3^.

Consequently, the 5-HT_1A_ receptor has been studied extensively^4–7^. Nevertheless, even fundamental questions as whether reduced or increased 5-HT_1A_ binding should be regarded as neuronal correlates of MDD have not been answered satisfactorily. More recent PET findings have provided some consistency for increased 5-HT_1A_ binding in drug naïve MDD compared to healthy controls, but these results may be dependent on the imaging methodology and specific regions analyzed^8–12^.

Within the encoding gene *HTR1A*, the polymorphism rs6265, also known as C(-1019)G, a common variation at the 1019 site upstream of the basal promoter area, has been associated with functional alterations in 5-HT_1A_ receptor signaling^13^. The more common C allele of this SNP allows binding of the transcriptional factor Deaf1 while the putative risk allele G blocks binding^14,15^. Deaf1 reveals cell specific effects in animal models, such as increasing cortical 5-HT_1A_ receptor binding while decreasing binding in the raphe^16^. Based on this molecular evidence, the G allele was studied in neuropsychiatric disorders and associated with MDD, bipolar disorder, suicide, as well as neuropharmacological drug response^17,18^. The association of the G allele with MDD was consistently replicated and confirmed in a meta-analysis a few years ago^19^. In addition, the G allele was associated with attenuated response to antidepressant drugs^20,21^.

Brain-derived neurotrophic factor (BDNF), critically involved in brain neuroplasticity, cell survival and axonal growth, has also been shown to influence the serotonergic system^22,23^. The val66met polymorphism, or rs6265, of the *BDNF* gene modulates BDNF activity by reducing proBDNF in carriers of the less frequent A or met allele^24^. The A allele has been associated with reduced resilience to stressful life events, aggression, anxiety and memory function. Consequently, it has been studied in a wide range of neuropsychiatric disorders, resulting in mixed findings for affective disorders, schizophrenia and neurodegenerative diseases^25^. Early studies reported the less common A allele to be more frequent in MDD than healthy controls, indicating a possible protective effect for A allele carriers^26^. On the contrary, more recent studies found the A allele to be associated with worse antidepressant treatment outcome, unfavorable clinical characteristics of MDD as psychotic features and suicidality, as well as increased anxiety^27,28^. These latter findings fit in well with the neuroplasticity hypothesis of depression and are backed up by animal models that demonstrated reduced BDNF trafficking in A allele carriers^29^. Ambiguities of the role of BDNF in affective disorders have been discussed for over a decade now. Nevertheless, meta analyses have not been able to disentangle the inconsistencies and reported mostly negative results for val66met and MDD^30,31^.

**Fig. 1:**
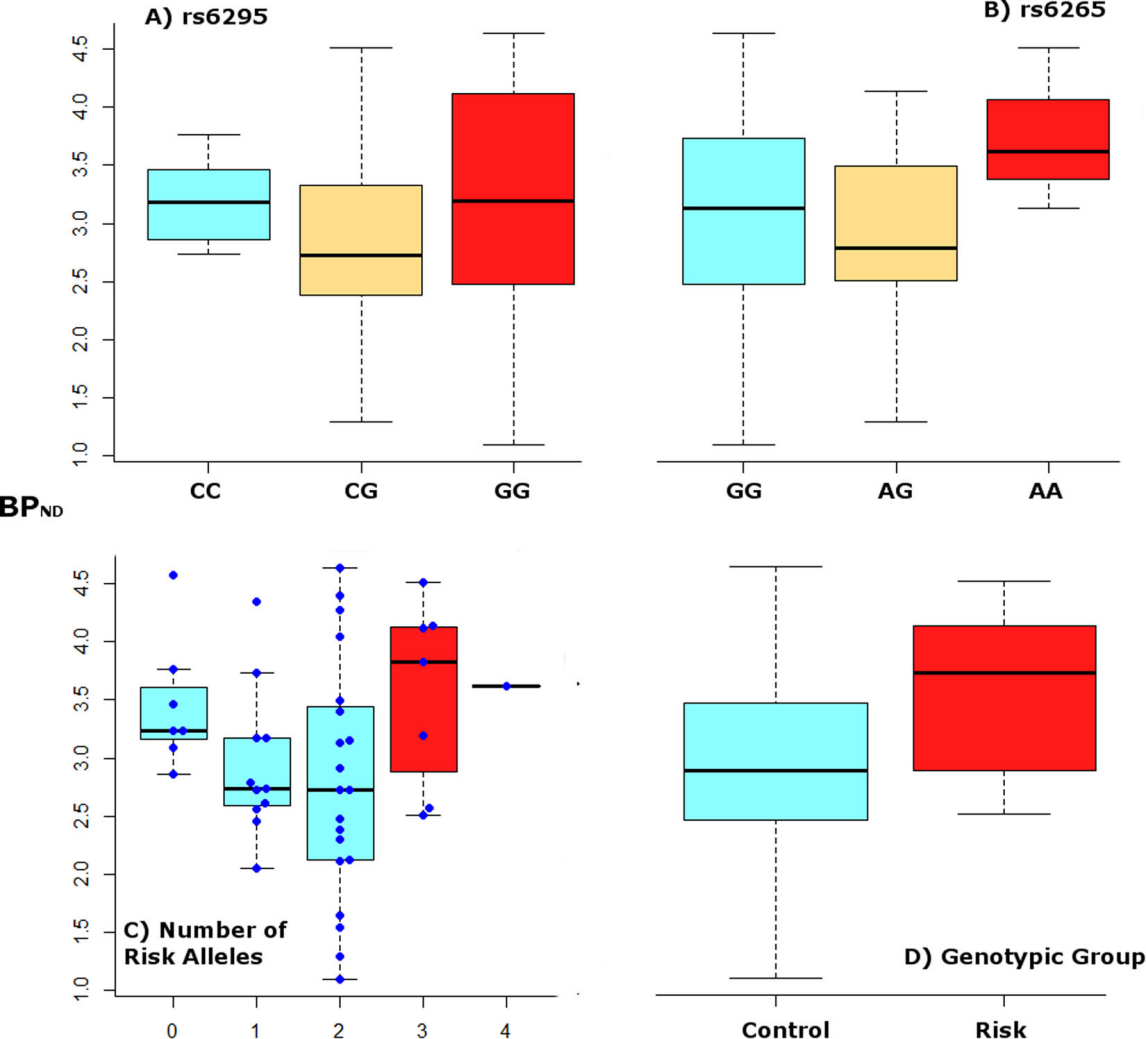
Boxplot for binding potential (BP_ND_), measured on the *y*-axis. Groups defined by risk alleles are colored red, those by control alleles are turquoise and heterozygote groups are beige. **a** Mean BP_ND_ is grouped by rs6265 genotype (AA = 3, AG = 17, GG = 26). **b** BP_ND_ is grouped by rs6295 genotype (CC = 10, CG = 19, GG = 13). **c** Groups are defined by the absolute number of risk alleles G of rs6295 and A of rs6265, ranging from 0 to 4 (0, *n* = 7; 1, *n* = 11; 2, *n* = 20; 3, *n* = 7; 4, *n* = 1). **d** BP_ND_ is grouped by genotypic group with control (*n* = 38) and risk phenotypes, the latter requiring at least 3 risk alleles (*n* = 8). The difference in mean BP_ND_ did not reach statistical significance for any comparison

**Fig. 2:**
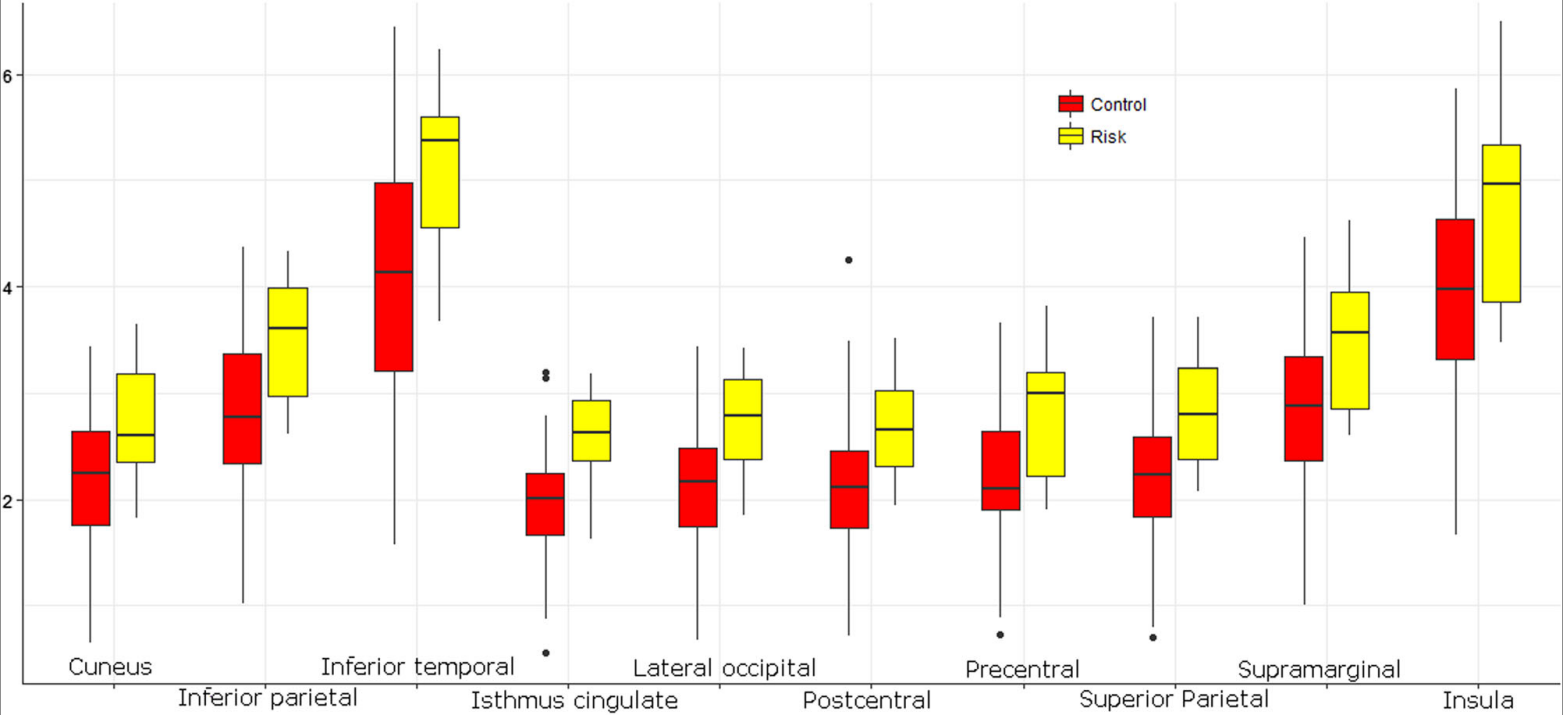
Average binding potential (BP_ND_) for all significant ROI according to post hoc ANOVA grouped by genotypic groups. Subjects with at least 3 putative risk alleles (*n* = 8) are colored red and are compared to the control sample (*n* = 38) portrayed in yellow. On the x-axis the 10 ROIs are listed, the *y*-axis shows binding potential (BP_ND_)

**Fig. 3:**
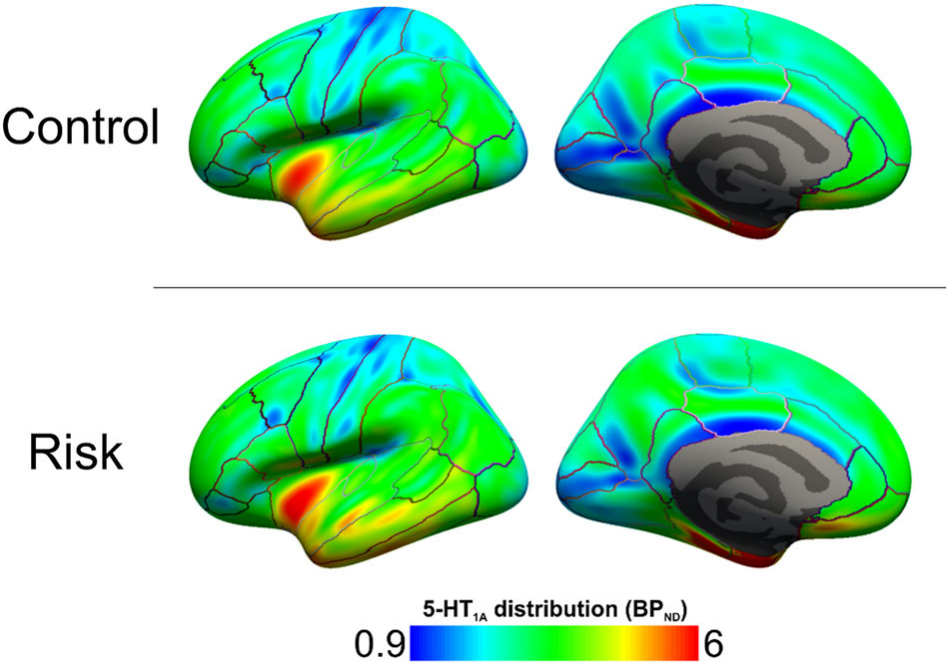
Brain map showing averaged cortical binding potential (BP_ND_) grouped by genotypic groups for all 32 brain regions analyzed. Subjects with at least 3 risk alleles (*n* = 8) are compared to the control sample (*n* = 38). The risk group is shows a higher average BP_ND_ in all regions

Few PET studies have investigated the effect of rs6295 and rs6265 on the serotonergic system (Tables 1 and 2). The G allele of rs6295 of *HTR1A* was initially associated with increased autoreceptor binding, indicated by elevated binding potential in the dorsal raphe nuclei of depressed patients. However, the same group reported no effects of rs6295 on 5-HT_1A_ receptor binding in a recent update with an expanded sample^9,11,32,33^. Exploiting the reciprocal effect of the rs6295 polymorphism on auto-receptors and heteroreceptors by introducing an alternative measure of non-displaceable binding potential (BP_ND_) normalized by the dorsal raphe binding, we recently demonstrated effects of rs6295 on 5-HT_1A_ receptor binding in patients with double G alleles^34^. However, in healthy subjects no effects have been demonstrated so far.

**Table 1:** Baseline characteristics for the genotypic risk and control groups, respectively

Baseline characteristics	Study sample (*n* = 46)		*p*-value
	Risk (*n* = 8)	Control (*n* = 38)	
rs6295 CC/C**G**/**GG**	0/4/**4**	10/15/**9**	
rs6265 GG/**A**G/**AA**	0/6/**2**	26/11/**1**	
Sex (male/female)	3/5	9/29	n.s.
Mean age ± SD	40 ± 13.59	43.82 ± 12.59	n.s.
Mean BP_ND_	3.56 ± 0.74	2.96 ± 0.88	n.s.

*Note*: Age and sex did not differ between groups, neither did mean nondisplaceable binding potential

*SD* standard deviation, *BP*_*ND*_ nondisplaceable binding potential

Risk alleles and respective counts in the study sample are indicated by bold values

**Table 2:** Mixed model results, interaction effects are marked with^x^

A) Mixed Model analysis	DF “*numerator”*	DF *“denominator”*	*F*-value	*p*-value
Group	1	44	3.2249	n.s.
ROI	32	1408	247.0292	<0.0001
ROI x genotype	32	1408	1.5577	0.048

B) Post Hoc ANOVA analyses			*F*-value	*p*-value
Cuneus			4.139	0.048
Inferior temporal gyrus			4.502	0.039
Inferior parietal gyrus			4.445	0.041
Insula			4.171	0.047
Isthmus cingulate gyrus			6.614	0.013
Lateral occipital gyrus			4.848	0.033
Postcentral gyrus			4.423	0.041
Precentral gyrus			4.684	0.036
Supramarginal gyrus			4.723	0.035
Superior parietal gyrus			5.441	0.024

*Note:* All mixed model *p*-values are corrected for multiple testing, post hoc analyses are uncorrected. For post hoc results, only significant ROI are shown

*ROI* region of interest, *DF* degree of freedom

Concerning rs6265 of *BDNF*, several targets of the serotonergic system were investigated, including serotonin transporter (SERT), 5-HT_1A_, and _2A_ receptor, as well as 5-HT_4_ receptor binding. The less common A allele resulting in methionine was suggested to decrease 5-HT_1A_ receptor binding measured by free plasma concentration binding potential (BP_F_) while other studies reported negative results based on BP_ND_^35–37^. Recently, the A allele was also associated with increased SERT binding in a large cohort of healthy subjects, while earlier studies reported gender-dependent lower binding in A-carriers or no differences^35,36,38^. No differences were found in 5-HT_2A_ receptor binding^39^. Finally, elevated 5-HT_4_ receptor binding was reported in A allele carriers by the same research group, suggesting a higher brain serotonin activity^40^.

There is evidence for interaction effects of functional genetic variations of BDNF and other genes in the serotonergic system^41–43^. A recent review examining the 5-HT_1A_ receptor in depression suggested interaction between *BDNF* and *HTR1A* as an important target for future PET studies^32^. Furthermore, the combination of the risk alleles of rs6265 and rs6295 was associated with treatment resistant depression in a clinical sample^44^. Regarding rs6265 and rs6295, only one PET study has investigated possible interactions of the two SNPs and reported negative findings^37^. Based on these findings we targeted the putative high risk polymorphisms for the 5-HT_1A_ receptor with PET imaging using [*carbonyl*-11 C]WAY-100635.

## Methods

### Subjects

All healthy subjects from three previously reported samples collected between 2004 and 2016 for who genotypes for rs6265 and rs6295 were available were pooled for this analysis^7,35,41,45,46^. Due to the lack of a patient sample of adequate size to investigate genetic interaction, only healthy subjects were considered. Consequently, 46 healthy subjects (34 female, 12 male) aged 18–65 (mean age 43.15 ± 13.08) were available for this cross-sectional neuroimaging study. The lack of single factor effects on 5-HT_1A_ receptor binding was shown before for overlapping cohorts of healthy subjects for rs6295 and rs6265^34,35^. However, surface-based results and particularly the interaction effects of rs6295 and rs6265 have not been previously published. Neuropsychiatric disorders were ruled out for all subjects using the Structured Clinical Interview for DSM-IV type disorders (SCID I + II). All participants underwent a physical and neurological examination including evaluation of clinical history, ECG, routine laboratory analysis, urinary drug, and pregnancy tests. Exposure to any neurotropic drugs or medication over lifetime was an exclusion criterion. All subjects gave written informed consent after receiving detailed oral information concerning the study procedures. The Ethics Committee of the Medical University of Vienna was involved in all studies relevant for this pooled sample and approved all study related procedures. Fo an overview of characteristics of the study sample, please see also Table 1.

### Genotyping

Genotyping procedures were described in previous publications^35,46^. In summary, Ethylene-Diamine-Tetraacetic-Acid (EDTA) blood samples of 9 ml were extracted from each subject and whole blood was used for DNA isolation with QiaAmp DNA blood maxi kit (Qiagen, Hilden, Germany). The iPLEX assay and the MassARRAY MALDI‐TOF mass spectrometer were used for genotyping, for details please see^47^. Identification of allele specific extension products and definition of genotypes was performed with Typer 3.4 Software (Sequenom, San Diego, CA). Quality requirements for genotyping were defined as an individual call rate above 80%, a SNP call rate over 99% and over 99% fit of genotyped CEU trios (Coriell Institute for Medical research, Camden, NJ) with the HapMap database.

### Radiochemistry and imaging procedures

The Division of Nuclear Medicine of the Department of Biomedical and Image‐guided Therapy of the Medical University of Vienna was responsible for all radiosynthetic procedures and provided the PET scanners (General Electric Medical Systems, Milwaukee, WI)^48^. The tracer [*carbonyl*-11C]WAY-100635 was used for all PET scans. Based on the current literature [*carbonyl*-11C]WAY-100635 is the best available radioligand for in vivo 5-HT_1A_ receptor quantification and shows favorable affinity and selectivity^49–51^.

The protocol for measurements required a 5‐min transmission scan using a retractable ^68^Ge rod source to achieve tissue attenuation correction. Next, dynamic emission scan was performed in 3‐D mode with mean injected doses of 309.76 ± 102.46 MBq and molar activity at time of injection of 281.21 ± 247.52 GBq/µmol. The radiochemical purity was above 95%. Data were reconstructed per volume via 35 transaxial sections (128 × 128 matrix) applying a filtered iterative back projection algorithm (FORE‐ITER). The spatial resolution was 4.36‐mm full‐width at half maximum 1 cm next to the center of the field of view (FOV). Magnetic resonance (MR) images were acquired for 20 of the participants using a 3‐Tesla Philips scanner (Achieva) and a T1-weitghted sequence, resulting in 1.56‐mm slice thickness and in plane resolution of 0.78 × 0.86 mm.

Subjects were placed with their head parallel to the orbitomeatal line guided by a laser beam system to ensure full coverage of the neocortex and the cerebellum in the FOV. A polyurethane cushion and head straps were used to minimize head movement and to guarantee a soft head rest during the whole scanning period.

### Data preprocessing

Freesurfer 6.0 (Harvard Medical School, Boston, USA; http://www.surfer.nmr.mgh.harvard.edu) was applied to reconstruct the cortical surface. T1-weighted MR images served as input whenever available, otherwise the ICBM 152-T1 template was used, after PET images were normalized to standard space, using a tracer-specific template^52^. To detect any mismatches in cortical surface reconstructions, all results were visually inspected. SPM12 (Wellcome Trust Centre for Neuroimaging, London, UK; http://www.fil.ion.ucl.ac.uk/spm) was used for motion correction. This was carried out by the realignment of frame images to median images resulting from a movement-free time period. Subsequently, the co-registration of the median PET images to individual MR images, as well as resulting surface reconstructions was performed. Finally, the motion-corrected dynamic images were combined with registration parameters to partition surface units in vertices.

### Kinetic modeling

Quantification of the cortical 5-HT_1A_ receptor distribution was computed with MATLAB 8.2 (https://www.mathworks.com) using the dynamic PET surface as input. In more detail, the multilinear reference tissue model (MRTM2) was applied to compute the cortical 5-HT_1A_ receptor availability (Ichise, M. et al. 2003). Thereby, the insular cortex was regarded as high-uptake region while the cerebellar white matter served as a reference region with putatively minimal 5-HT_1A_ receptor concentration^51^. Subsequently, 32 cortical ROI were delineated based on the Desikan-Killiany atlas^53^.

### Statistical analysis

The statistical software “R” was used for all investigations (cran.r-project.org). Differences between two customized genotypic groups were compared. Specifically, within rs6295 of *HTR1A*, the G allele has been demonstrated to cause transcriptional dysregulation leading to altered 5-HT_1A_ receptor binding. Thereby, transcriptional effects showed linear increase with the number of G copies^16^. On the other hand, the A allele of *BDNF* rs6265 decreases proBDNF levels, disrupting BDNF pathways and putatively also affecting 5-HT_1A_ receptor binding. Therefore, we compared high-risk individuals with at least three risk alleles within the two SNPs, including either homozygote subjects for G allele of rs6295 showing at least one A allele of rs6265 or homozygote subjects for A allele of rs6265 showing at least one G allele of rs6295. All other genotypic variations were included in the control group. This decision was based on positive findings in combined risk allele carriers in a clinical sample of treatment resistant depression and inconsistent results for imaging studies when either SNP was considered alone^34,35,37,44^. While an increase of effect with the number of unfavorable alleles was reported in preclinical studies, previous PET studies could not substantiate these findings in either patient or healthy cohorts^16,32^. We considered on one hand the small sample sizes available for most PET studies, as well as various compensatory mechanisms extenuating small effects in healthy subjects, and on the other hand the rarity of the most unfavorable genotype with two risk alleles for both SNPs. Thus, comparing subjects with at least three risk alleles to the rest was the preferred approach.

Differences of BP_ND_ between genotypic groups were investigated with linear mixed model as included in the “lmne” package of “R” in a ROI based approach^54^. Subject served as the random factor and genotypic group, ROI and their interaction served as fixed factors. Sex and age did not affect the model and were hence excluded. In total 32 surface based ROIs were integrated in the model based on the Desikan atlas. For mixed model results a threshold of *p* < 0.05 after Bonferroni correction (for number of models, main and interaction effects) was set for significance. For the post hoc analyses for specific ROIs with ANOVA, a *p*-value threshold of 0.05 was determined.

## Results

Risk and control genotype groups did not differ in age or sex. Genotypes for rs6295 and rs6265 were in Hardy Weinberg equilibrium (*p* < 1). The risk group showed overall increased BP_ND_ (mean BP_ND_ 3.56 ± 0.74 vs. 2.96 ± 0.88; *F* = 3.225, *p* = 0.08). Mean BP_ND_ for genotypes of rs6265 and rs6295, by number of risk alleles and for the risk and control groups is portrayed in Figure 1.

As expected, ROI showed significant effects in the mixed model (*F* = 247.03, *p* < 0.001, Bonferroni corrected for the number of models and effects). Furthermore, an interaction effect could be demonstrated for ROI and genotype (*F* = 1.567, *p* = 0.048, Bonferroni corrected for the number of models and effects). See also Table 2, section A for mixed model results.

Post hoc analyses with ANOVA were used to identify genotypic effect within specific ROIs and a *p*-value of 0.05 was regarded as significant. Differences between the risk and control genotypes could be demonstrated in 10 regions, as portrayed in Table 3. These were the cuneus, supramarginal, superior parietal, lateral occipital, isthmus cingulate, inferior temporal, inferior parietal, insula, postcentral and precentral gyrus. See also Table 2, section B, and Figure 2 for post hoc results. For a brain map depicting differences in mean BP_ND_ for all cortical ROI between risk and control groups please refer to Figure 3.

## Discussion

Investigating a large sample of 46 healthy individuals with [*carbonyl*-11C]WAY-100635, we observed higher 5-HT_1A_ receptor binding by an average of 17% in the risk group with a genotype of 3 or more putative risk alleles of the two SNPs, rs6265 and rs6295, combined. Our results substantiate the evidence of *BDNF* and *HTR1A* polymorphisms val66met and C(-1019)G affecting the serotonergic system in the healthy brain. Increased 5-HT_1A_ BP_ND_ is a well-published phenotype of depressed patients^9–11,33^. Thus, healthy subjects with a risk genotype based on epistasis of rs6265 and rs6295 show similarities with imaging results in MDD.

Both polymorphisms rs6295 and rs6265 show molecular mechanics, which allow them to directly impact the serotonergic system.

For rs6295, the transcription factor Deaf1 is blocked by the G allele at the transcription site, leading to significantly increased 5-HT_1A_ receptor binding in the raphe nuclei but decreased cortical binding in knockout mouse models^14,55^. However, in vivo evaluation of 5-HT_1A_ receptor binding measured by PET in humans could not confirm these mechanisms, as only differences in the dorsal raphe have been reported. A correlation of BP_F_ with the number of G alleles could be observed in 2006 and was initially replicated in 2011^9,33^. However, a final evaluation with an expanded sample and refined statistics led the same group to the conclusion that no functional effects can be observed in humans with or without MDD^8^. Concerning the heteroreceptors, an overall but insignificant increase in 5-HT_1A_ receptor binding in G allele carriers was observed in the studies described above in cortical areas and in patients with bipolar depression in amygdala and hippocampus^32,56^. These findings were in line with elevated 5-HT_1A_ receptor binding in depressed subjects demonstrated by some studies, however, did not fully converge to the molecular mechanisms reported from animal models regarding rs6295^9,11,12,33^. On the other hand, we did not observe significant differences in BP_ND_ between rs6295 genotype, unless a transformed outcome parameter BP_Div_ was used, normalizing BP_ND_ by individual raphe binding potential and therefore exploiting subtle but opposite effects on raphe and projection areas^34^.

There is increasing evidence for direct interactions between serotonin and BDNF^57,58^. Synthesized as a pre-proneurotrophin, proBDNF is dependent on the removal of the pre-region and requires cleavage by proteases to reach the active form BDNF. The polymorphism rs6265 is especially interesting as the A allele has been demonstrated to reduce proBDNF trafficking and therefore decrease BDNF activity in cell models^24^. Effects seem to be predominant in the central nervous system as cortical neurons and neurosecretory cells, but not endothelial and vascular smooth muscle cells, demonstrated decreased BDNF secretion^59^. These mechanisms could also be observed in animal models, where the homozygous A allele genotype led to anxiety phenotypes^29^. Furthermore, PET studies have been conducted with regards to rs6265 and SERT, 5-HT_1A_, 5-HT_2A_, and 5-HT_4_ receptor binding. Just recently, reduced 5-HT_1A_ receptor binding was reported in A allele carriers while two previous studies reported no effect of rs6265 genotype^35–37^. Furthermore, the largest study so far reported increased SERT binding in A allele carriers, while originally lower binding or absence of genotype effects was reported^35,36,38^. The same group also demonstrated an increase of 5-HT_4_ receptor binding in A allele carriers, suggesting reduced serotonin levels^40^.

Taken together, these data support functional effects of rs6265 and rs6295 on the serotonergic system, however, the implications and extent of these effects are not yet clear. In synopsis of our studies, we found increased cortical 5-HT_1A_ receptor binding in the risk group, but no effect when either SNP was analyzed separately^34,35^. This interaction effect and the lack of a linear increase of BP_ND_ with the number of risk alleles points towards a possible epistasis between *HTR1A* and *BDNF*. However, we cannot rule out a simply additive effect of the risk alleles on 5-HT_1A_ binding potential as both SNPs may show significant associations considered separately in sufficiently large sample. In any case, our results indicate healthy controls with combined genetic risk show increased 5-HT_1A_ receptor binding. This is concordant with alterations in depressed patients described by preclinical and imaging findings^32,60^. While the molecular mechanisms of depression are still not sufficiently understood, most of the currently prescribed antidepressant agents target the serotonergic system by blocking the SERT and putatively desensitizing the 5-HT_1A_ autoreceptors while increasing postsynaptic 5-HT_1A_ signaling^14^. In addition, increase of BDNF has been attributed to various antidepressant treatments, including ketamine and ECT^61,62^. Thus, the two functional polymorphisms rs6265 and rs6295 regulating 5-HT_1A_ signaling and BDNF trafficking may be of relevance for most currently applied antidepressant treatments.

Most importantly, our results differ from the only other PET study on 5-HT_1A_ receptor binding in healthy controls with regards to rs6265 and rs6295, showing decreased binding in A allele carriers but no interaction effect with rs6295^37^. Contrary to previous studies, we focused on cortical ROI due to application of surface based modeling with FreeSurfer. Decisive advantages of this approach have been highlighted recently, most importantly decreasing intersubject variance, a major limiting factor in PET studies investigating the 5-HT_1A_ receptor^63,64^. Surface-based modeling can reduce bias by sustaining cortical geometry, resulting in the gray matter signal being less contaminated with white matter and cerebrospinal fluid^65^.

Besides these differences, divergent findings have partly been explained by methodological variation in PET studies. Different approaches to calculate the concentration of the 5-HT_1A_ receptor have been applied, most notably BP_ND_ and BP_F_. These refer to the ratio of specifically bound tracer in tissue to either the concentration of free tracer in plasma (BP_F_) or to the concentration of non-displaceable tracer in tissue (BP_ND_), thereby aiming to attain the best estimate of the number of available binding sites, B_avail_^66^. BP_ND_ does not require arterial blood sampling and is therefore less invasive. However, BP_F_ has been demonstrated to be more favorable as it is independent of a reference region, which could bring bias to the outcome measure. There is insufficient data on direct comparison of BP_ND_ and BP_F_. One study has shown effects may be different or even opposite, depending on the applied BP variant^33^. Differences were explained by minimal but confounding binding in the reference region, which may also be affected by the genetic polymorphisms investigated. As a control measure, we compared cerebellar white matter time activity curve counts registered during PET measurement between risk and control groups. No significant differences were observed (*p* = 0.47, *t* = 0.729), indicating that the reference tissue model did not compromise the results due to different binding between genotypic groups in the cerebellum. However, as no direct comparison of the methods was possible, we cannot rule out bias due to these methodological differences.

Another limitation is that our sample has been collected over a decade and is pooled from different PET studies with [*carbonyl*-11C]WAY-100635. As PET is resource intensive and genetic investigations demand larger samples than usually collected for PET studies due to small effect sizes and stratification by genotypic groups, pooling is often necessary in imaging genetics. Nevertheless, we are confident our results are not significantly biased by pooling procedures. All subjects underwent the same screening procedures concerning somatic and neuropsychiatric disorders and drug naivety, and were measured with the same PET scanner. However, the sample size of 46 subjects is still small and our results must be interpreted as exploratory unless validated in larger, independent samples. It may also be relevant that our sample was skewed towards female sex with roughly 74% female subjects. While we did not observe significant differences between average BP_ND_ of male and female subjects (mean BP_ND_ = 3.15 and 3.01, respectively; *p* > 0.05), effects of as progesterone and testosterone on 5-HT_1A_ binding have been reported previously^67,68^. On the other hand, we did not observe an impact of sex or hormone replacement therapy on 5-HT_1A_ binding in previous PET studies, indicating a less pronounced effect of sex hormones^7,69^.

Apart from these limitations, some other considerations should be discussed.

First, the epigenetic contribution and methylation status have been neglected. The importance of epigenetics, which is still not fully established in neuropsychiatric research, has been consistently demonstrated in the last years. Epigenetic differences explained discrepancies within MDD in monozygotic twins and several methylation markers were suggested as predictors of MDD^70,71^. There is also evidence that 5-HT_1A_ receptor availability is regulated by a Sp4 site, prone to stress induced hypermethylation, potentially directly impacting 5-HT_1A_ receptor binding PET studies^72^. Consequently, grouping solely by genotype without considering methylation-induced inactivation of the target SNPs may be insufficient for some subjects. However, despite strong recommendations to examine methylation in genetic investigations, there have hardly been PET studies accounting for epigenetic effects so far^73^.

Another contributor to divergent findings in imaging genetics could be allosteric heteroreceptor complexes^74^. 5-HT_1A_ receptors have been demonstrated to form heteroreceptor complexes with various G-protein coupled receptors^75^. Tyrosine kinase receptors and tumor necrosis factor receptors relevant to BDNF have also been shown to form heteroreceptor complexes^76^. The lack of findings in direct support of the molecular mechanisms of rs6295 may be explained by FGFR1-5-HT_1A_ receptor complexes in the raphe nuclei that can disrupt the negative feedback of autoreceptors and eventually lead to 5-HT_1A_ receptor boosting effects in cortical areas^77^. Even though two studies targeting SERT and 5-HT_1A_ receptor binding with regards to rs6265 reported no interaction effects, implementation of different serotonergic targets in a multivariate model could be mandatory to disentangle the complex genetic scaffoldings of the serotonergic system. However, methodological advances, economizing scanning time and radiation exposure, will probably be necessary to make these models viable^78,79^.

In summary, using PET imaging and [*carbonyl*-11C]WAY-100635, we provide further evidence the two functional SNPs rs6265 of *BDNF* and rs6295 of *HTR1A* impact 5-HT_1A_ receptor binding. Importantly, our results indicate these SNPs do not exert influence on their own, but rather through epistasis, as only subjects with three or more risk alleles showed increased 5-HT_1A_ BP_ND_. Thus, we propose that epistasis between *HTR1A* and *BDNF* is a control element of the serotonergic system and may be involved in neuropsychiatric disorders as depression. As only healthy subjects were included in this analysis, further investigations in depressed patients are needed to clarify the role of this interaction in MDD. Keeping the limitations in mind, we cannot rule out independence of our findings from methodological issues, such as binding potential computation or the radiotracer used. Epistasis between *HTR1A* and *BDNF* may be an important contributor to affective disorders and potentially could become a target for diagnosis and treatment. Our findings are encouraging to further investigate interactions of rs6265 and rs6295 in larger cohorts as implementation of epigenetics and allosteric effects are necessary to fully determine the role of these SNPs in the serotonergic system.

## Electronic supplementary material

Supplement
