# Correction: Epistasis of HTR1A and BDNF risk genes alters cortical 5-HT1A receptor binding: PET results link genotype to molecular phenotype in depression

**DOI:** 10.1038/s41398-019-0583-6

**Published:** 2019-10-04

**Authors:** Alexander Kautzky, Gregory M. James, Cecile Philippe, Pia Baldinger-Melich, Christoph Kraus, Georg S. Kranz, Thomas Vanicek, Gregor Gryglewski, Annette M. Hartmann, Andreas Hahn, Wolfgang Wadsak, Markus Mitterhauser, Dan Rujescu, Siegfried Kasper, Rupert Lanzenberger

**Affiliations:** 1 Department of Psychiatry and Psychotherapy, Medical University of Vienna, Wien, Austria; 2 Division of Nuclear Medicine, Department of Biomedical Imaging and Image-guided Therapy, Medical University of Vienna, Wien, Austria; 3 University Clinic for Psychiatry, Psychotherapy and Psychosomatic, Martin-Luther-University Halle-Wittenberg, Halle, Germany; 4 Center for Biomarker Research in Medicine (CBmed), Graz, Austria; 5 Ludwig Boltzmann Institute Applied Diagnostics, Vienna, Austria

**Keywords:** Depression, Molecular neuroscience, Genomics

**Correction to: Translational Psychiatry**

10.1038/s41398-018-0308-2 published online 16 January 2019

In the original Article, co-author Professor Andreas Hahn was not included in the author list. This has been corrected in the XML, PDF and HTML versions of this Article.

