# Supplementary material for: Epistasis of HTR1A and BDNF risk genes alters cortical 5-HT1A receptor binding: PET results link genotype to molecular phenotype in depression
Source: Transl Psychiatry. 2019 Jan 16;9:5. doi: 10.1038/s41398-018-0308-2 (PMC6341100; doi:10.1038/s41398-018-0308-2)
Supplement: Supplementary file 1 — Supplement [file 41398_2018_308_MOESM1_ESM.pdf]

Supplementary Data

Table of regions of interest as numbered in Supplementary eFigure 1 to 3

1. Caudal Anterior Cingulate
2. Caudal Middle Frontal
3. Cuneus
4. Entorhinal
5. Fusiform
6. Inferior Parietal
7. Inferior Temporal
8. Isthmus Cingulate
9. Lateral Occipital
10. Lateral Orbitofrontal
11. Lingual
12. Medial Orbitofrontal
13. Middle Temporal
14. Parahippocampal
15. Pars Opercularis
16. Pars Orbitalis
17. Pars triangularis
18. Pericalcarine
19. Postcentral
20. Posterior Cingulate
21. Precentral
22. Precuneus
23. Rostral Anterior Cingulate
24. Rostral Middle Frontal
25. Superior Frontal
26. Superior Parietal
27. Superior Temporal
28. Supramarginal
29. Frontal Pole
30. Temporal Pole
31. Transverse Temporal
32. Insula

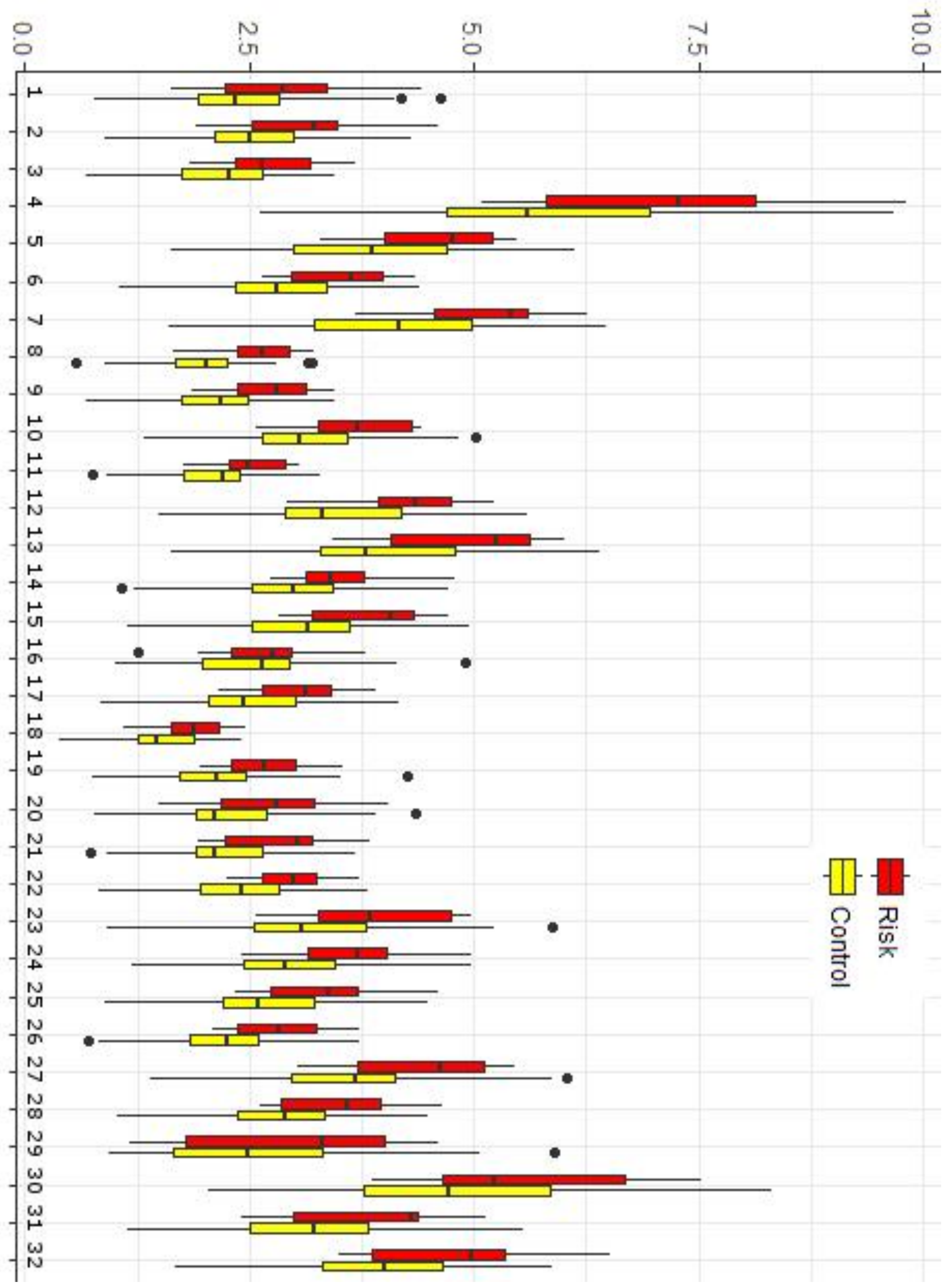

Supplementary eFigure 1. Average binding potential (BP_{ND}) for all ROI grouped by genotypic groups. On the x-axis all ROIs are listed, the y-axis shows binding potential (BP_{ND}). The control group is colored yellow, the risk group red.

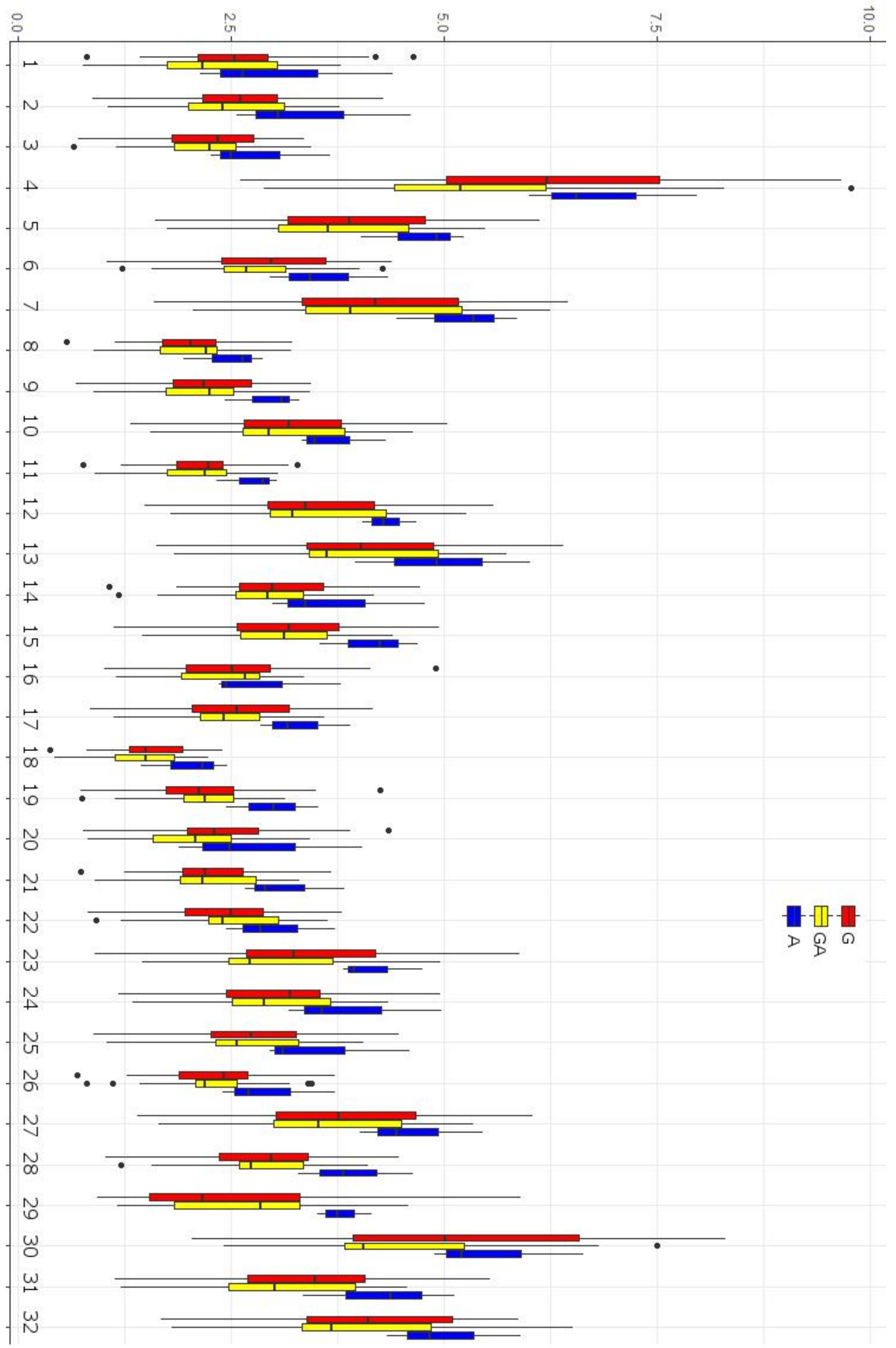

Supplementary eFigure 2. Average binding potential (BP_{ND}) for all ROI grouped by rs6265 genotype. On the x-axis all ROIs are listed, the y-axis shows binding potential (BP_{ND}). Double G carriers are colored red, heterozygotes yellow and the risk group blue.

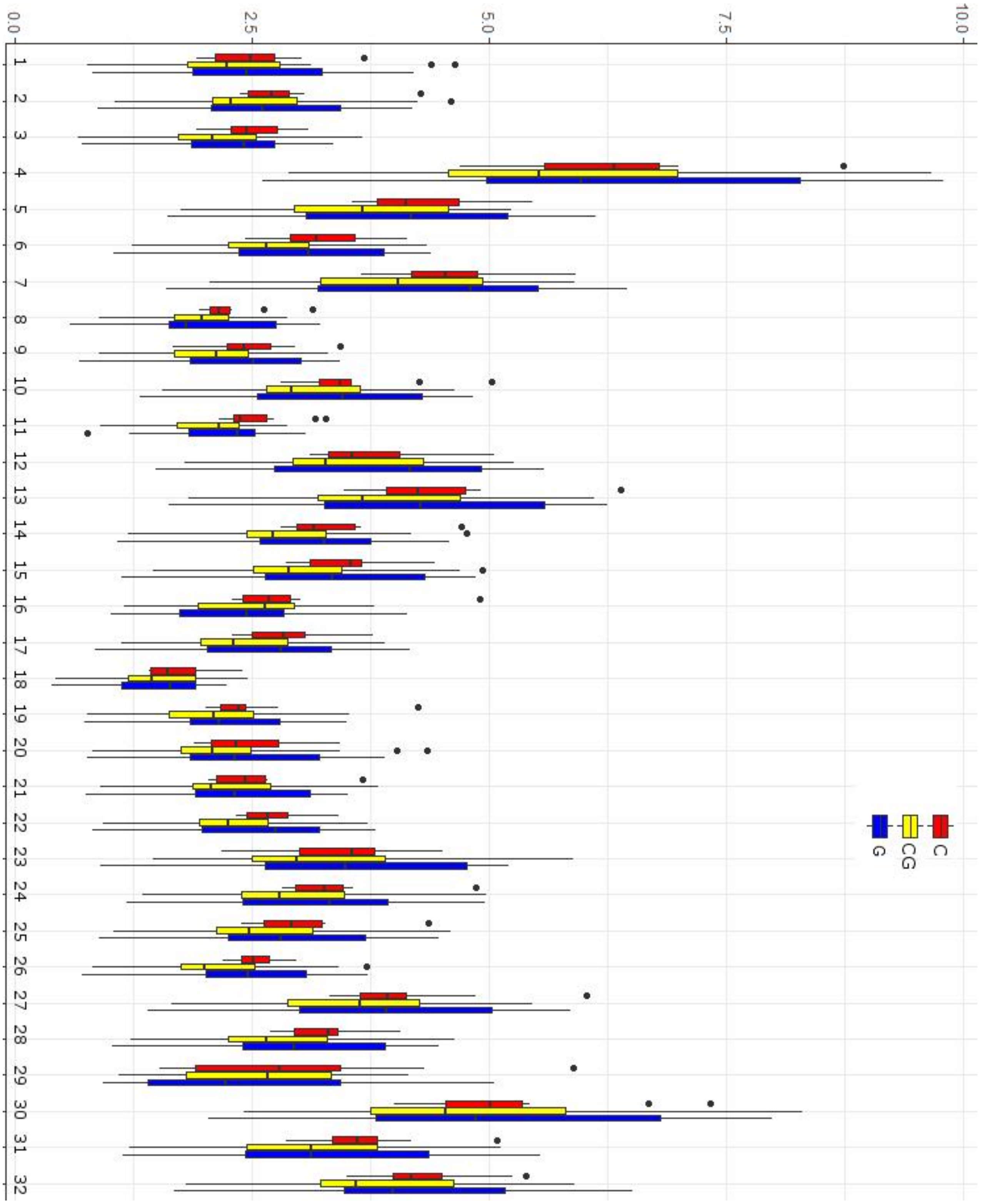

Supplementary eFigure 3. Average binding potential (BP_{ND}) for all ROI grouped by rs6295 genotype. On the x-axis all ROIs are listed, the y-axis shows binding potential (BP_{ND}). Double C carriers are colored red, heterozygotes yellow and the risk group blue.
